# Liquid Biopsy of Non-Plasma Body Fluids in Non-Small Cell Lung Cancer: Look Closer to the Tumor!

**DOI:** 10.3390/cells9112486

**Published:** 2020-11-16

**Authors:** Lucile Durin, Anne Pradines, Céline Basset, Bryan Ulrich, Laura Keller, Vincent Dongay, Gilles Favre, Julien Mazieres, Nicolas Guibert

**Affiliations:** 1 Pulmonology Department, Hôpital Larrey, University Hospital of Toulouse, 31059 Toulouse, France; durin.l@chu-toulouse.fr (L.D.); dongay.v@chu-toulouse.fr (V.D.); mazieres.j@chu-toulouse.fr (J.M.); 2 Cancer Research Centre of Toulouse (CRCT), Inserm, National Scientific Research Centre (CNRS), 31100 Toulouse, France; Pradines.Anne@iuct-oncopole.fr (A.P.); Keller.Laura@iuct-oncopole.fr (L.K.); favre.gilles@iuct-oncopole.fr (G.F.); 3 Medical Laboratory, Claudius Regaud Institute, Toulouse University Cancer Institute (IUCT-O), 31100 Toulouse, France; 4 Cytology Department, Toulouse University Cancer Institute (IUCT-O), 31100 Toulouse, France; basset-leobon.c@chu-toulouse.fr; 5 Emory University School of Medicine, Atlanta, GA 30322, USA; Bryan_Ulrich@DFCI.HARVARD.EDU; 6 University of Toulouse III—Paul Sabatier, 31062 Toulouse, France

**Keywords:** genotyping, targeted therapy, screening, urine, fine-needle aspiration, cerebrospinal fluid, liquid biopsy, circulating tumor DNA, lung cancer

## Abstract

Liquid biopsy is a rapidly emerging field due to an increasing number of oncogenic drivers and a better understanding of resistance mechanisms to targeted therapies in non-small cell lung cancer (NSCLC). The sensitivity of the most widely used blood-based assays is, however, limited in particular in cases of low tumor volume where shed of tumor-derived material can be limited. A negative result thus requires biopsy confirmation using minimally invasive sampling procedures that can result in small specimens, which are often not suitable for genotyping. Liquid biopsy is not limited to plasma, and tumor DNA circulating in other body fluids such as urine, pleural fluid, cerebrospinal fluid, or cytology specimen-derived supernatant can be exploited. In comparison to cell blocks, these fluids in close contact to the tumor may contain a more abundant and less analytically demanding tumor DNA. In this review, we discuss the potential applications of circulating tumor DNA derived from cytology samples in NSCLC, from early stage (screening, nodule characterization) to metastatic disease.

## 1. Introduction

Mechanisms of oncogenesis in lung cancer have been largely deciphered over the past 20 years. Lung adenocarcinoma, unlike other histological subtypes, can now be considered as a cluster of discrete molecular subtypes, the majority being defined by a single alteration of an oncogenic driver. In addition to *EGFR* and *KRAS* mutations and *ALK* rearrangements, new molecular targets, such as *BRAF*, *MET*, or *HER2* mutations, and *ROS1*, *NTRK*, and *RET* rearrangements, have been recently highlighted. Multiplex genotyping and high-throughput genomic profiling by next-generation sequencing (NGS) is thus increasingly refining molecular diagnoses [1]. The evolution of tumors bearing a molecular alteration is usually dependent on a single mechanism following the principle of oncogenic addiction, which has been described as the dependence of tumor cells on the specific activity of an activated oncogene [2]. These tumors respond to genotype-directed therapies but inevitably progress usually through the emergence of genomic alterations that confer resistance to first-line targeted agents, requiring iterative assessment of the molecular profile of the tumor. In parallel, sampling methods are also evolving. There is currently a paradox between the need to obtain a significant amount of tumor tissue to test an increasing number of biomarkers and the development of bronchoscopic minimally invasive techniques, resulting in small tissue samples with limited amounts of DNA [1]. Bronchoscopy currently constitutes the preferred approach for tumor sampling as it is less invasive than radio-guided biopsies [3]. It is however frequent (10 to 20% of cases) [4,5] that these cytologic samples, when only the cell block is considered, are rejected for genotyping after time consuming processing steps because of limited tumor content.

Liquid biopsy, and in particular plasma circulating tumor DNA (ctDNA) genotyping to detect driver and resistance mutations, is a rapidly emerging field in non-small cell lung cancer (NSCLC) [6]. While tissue only offers a snapshot of the tumor at a given time and location, liquid biopsy has the potential to overcome both spatial and temporal tumor heterogeneity and can non-invasively interrogate the molecular landscape of a tumor, taking into account different clones present within all metastatic sites, and can follow subclonal evolution through iterative blood draws. However, the most sensitive cell-free DNA (cfDNA) genotyping platforms still have a sensitivity of only 70–80% for advanced diseases [7,8,9] and below 50% for early stages [6,10], such that a negative result requires biopsy confirmation. This poses a clinical challenge because false negative plasma genotyping is usually associated with limited metastatic spread and lower tumor burden [9], and biopsy of these patients may be more challenging. Blood sampling strategies at the tumor draining vein could be a solution to increase recovery rate, however such a strategy has so far only been applied to circulating tumor cell (CTC) detection and necessitates additional steps during surgery [11,12].

However, liquid biopsy is not limited to plasma. Tumor nucleic acids floating (alone or in exosomes) in other body fluids such as urine, pleural fluid, cerebrospinal fluid, or cytology specimen-derived supernatant can be exploited.

**Figure 1:**
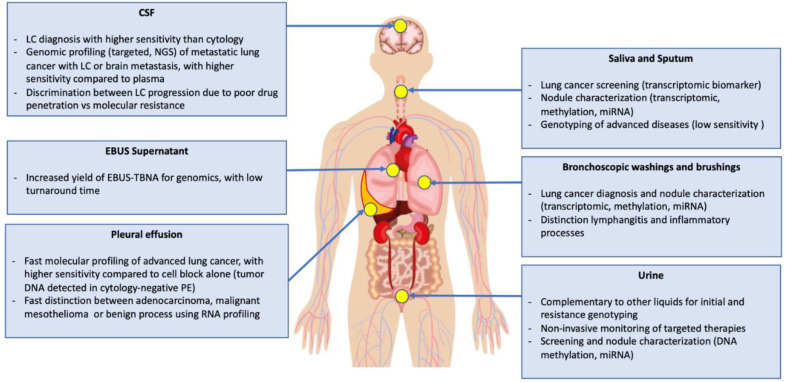
Easily available body fluids and their potential applications. BW: bronchial washing, PE: pleural effusion, CSF: cerebrospinal fluid, LC: leptomeningeal carcinomatosis, EBUS-TBNA: endobronchial ultrasonography transbronchial needle aspiration, NGS: next generation sequencing.

A fluid close to a tumor/metastasis may contain more abundant and less diluted tumor DNA (tDNA) with higher concentrations/mutant allelic fraction (MAF), derived from active secretion and/or cell death, compared to what can be isolated from the bloodstream. Non-invasive collection of multiple fluids could be complementary and increase sensitivity compared to plasma alone and may better characterize spatial tumor heterogeneity. We report herein the main “non-blood” biological fluids that have been studied for liquid biopsy tests in the field of NSCLC and their potential advantages (summarized in Table 1 and Figure 1). We studied the main publications reported in journals indexed on PubMed (using the terms: Pleural effusion cfDNA, urine cfDNA, CSF cfDNA, ascitic fluid cfDNA, supernatant cfDNA, brushings cfDNA, washings cfDNA) on the topic in the past years.

## 2. Fine-Needle Aspiration (FNA) Supernatant

The supernatant of fine-needle aspiration (FNA) specimens (Computed tomography (CT)-guided or EBUS-TBNA (endobronchial ultrasonography transbronchial needle aspiration) in the context of NSCLC) is usually discarded. However, it is now clear that this supernatant is rich in cell free DNA. In patients with pancreatic cancer, the yield of FNA supernatant matches, and in a subset of patients, exceed cellblock [13]. Roy-Chowdhuri et al. reported in FNA supernatant mean and median DNA yields of 445 ng and 176.4 ng (i.e., much higher than what is usually found in plasma), respectively. Tumor-associated mutations were detected (via NGS) in all 25 FNAs from patients with solid tumors and none of the 10 benign controls [14].

**Figure 2:**
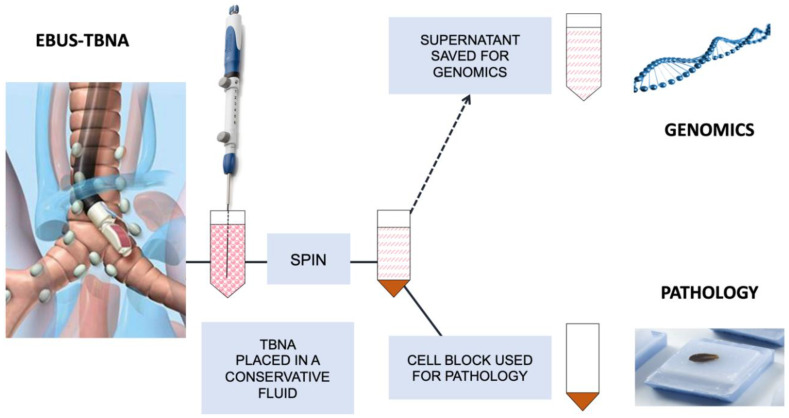
Alternative handling of cytology specimens for genomics using Fine-Needle Aspiration (FNA) supernatant: example of EBUS-TBNA. EBUS-TBNA: endobronchial ultrasonography transbronchial needle aspiration.

In the context of lung cancer, several studies have also demonstrated the feasibility and accuracy of NGS-based genotyping of cfDNA derived from FNA supernatant [14,15,16,53]. In particular for EBUS-TBNA, in which a specimen of low tumor cell content is rejected from genotyping in up to 20% of cases [17], mutations of interest could be detected in all supernatant-derived cfDNA from a 17 patient cohort [15]. Genomics is usually performed after conventional time-consuming diagnostic steps on the cell block (e.g., tumor adequacy review, formalin fixed paraffin embedded (FFPE) preparations, immunostaining). Supernatant, an immediately available biospecimen, could significantly improve the overall yield of these pauci-cellular samples for genomics and decrease the turnaround time [18]. This is particularly appealing in the context of tyrosine kinase inhibitors (TKI) resistance where histology is less needed since, with the exception of the rare small cell transformation, most resistance mechanisms are genetic alterations. The example of a potential alternative handling of EBUS-TBNA is summarized in Figure 2.

## 3. Bronchoscopy Cytology Specimen Supernatant

With the increase in lung cancer screening, the management of peripheral nodules is becoming a highly prevalent and challenging situation for pulmonologists. Because most nodules are benign and biopsies are difficult and dangerous (CT-guided biopsies are complicated with pneumothorax in more than 20% of cases), less invasive approaches are urgently needed to discriminate benign from malignant nodules. Sensitivity of plasma genotyping remains poor for early stage NSCLC because of inconsistent/low DNA shed [10]. Bronchoscopy, a minimally invasive procedure, appears to be a good compromise. Cytology examination of bronchoalveolar lavage has poor sensitivity to detect lung cancer but its yield could be improved using new biomarkers, such as exosome-derived miRNA [19] or free DNA methylation (e.g., targeting SHOX2) [20]. Ruy et al. demonstrated that DNA extracted from bronchial washing (BW) supernatant has potential to increase the yield of BW for early-stage lung cancer detection [21].

The free DNA derived from bronchoscopy brushings and washings has also been investigated for advanced lung cancer genotyping by using a TaqMan PCR assay limited to common *EGFR* mutations. The sensitivity reached 88% across 74 specimens with no false positives [22]. *EGFR* genotyping in bronchoalveolar lavage fluid could be improved by the use of extracellular vesicles (EV) as demonstrated in a study of 137 patients [23]. The sensitivity and specificity of EV-based *EGFR* genotyping were 76% and 87%, respectively, with high sensitivity irrespective of the stage (79% for stage I, 100% for stage II, 74% for stage III, and 92% for stage IV).

New bronchoscopic approaches to better target peripheral nodules have been developed such as electromagnetic navigation or radial EBUS, but these samples are sometimes of low tumor content and sensitivity is low for the small lesions (below 70% for nodules < 20 mm) [3]. Following a similar approach as the one reported in the previous chapter, it is very likely that the supernatant derived from the cytology samples collected during these procedures (brushing, rinses) are of high free floating DNA content and could be used to help characterize nodules (using DNA methylation analysis, for example).

## 4. Pleural, Pericardial, and Ascitic Fluids

Pleural, pericardial, and ascitic fluids are ultrafiltrates from the blood deprived of peripheral blood cells but that can be enriched in cfDNA. Malignant pleural effusion (MPE) is a common complication of lung cancer. Pleural effusion (PE) supernatant contains cell free tumor DNA (PE-cfDNA) with high MAF (comparable to tissue). A study of 102 NSCLC patients showed a good correlation between supernatant-cfDNA and cell block molecular profile (NGS) in MPE [24]. Tong et al. recently demonstrated in 63 lung cancer patients that PE-cfDNA outperforms plasma and cell block for genotyping, with some samples being cytologically negative but rich in free-floating tumor DNA [25]. Reliable detection of *EGFR* mutations in cytology-negative pleural effusion has been demonstrated in another study [26]. Thus, NGS genotyping of PE-cfDNA appears to be a very appealing approach in cases where no tissue is left. Additionally, it may have a lower turnaround time and a higher sensitivity in cases with adequate tissue. Moreover, tumor mutational burden in PE-cfDNA and in tumor tissue DNA are well correlated [27]. This rich source of tumor DNA can be used to quickly detect driver and resistance *EGFR* mutations using digital droplet PCR (ddPCR), with high concordance between supernatant and cell pellet [28,29]. Other supernatant-derived material can be used to provide genomic information (*EGFR* T790M mutations in particular [30]), such as extracellular vesicle-derived DNA (EV-DNA). DNA concentration in EV-DNA extracted from pleural effusion supernatant was significantly higher than that of cfDNA. Moreover, EV-DNA demonstrated 100% agreement with tissue for *EGFR* genotyping, with higher specificity compared to cytology [30].

After surgery for early stages, liquid biopsy of pleural effusion could help discriminate a metastatic PE from a benign process using RNA profiling (miR-200 and LCN2 expression) [31]. miR-130A quantification could help distinguish lung adenocarcinoma and malignant mesothelioma [32].

Similarly to pleural fluid, metastatic ascites contains free-floating DNA that can be used for mini-invasive and fast initial or resistance genotyping, as demonstrated by the detection of copy number variations (CNVs) in cancer-associated genes in a small series of 6 metastatic non lung cancer patients [33]. Another prospective study has reported that ascitic fluid in patients with peritoneal carcinomatosis (*n* = 8, among other liquids) is more sensitive than plasma for the detection of clinically relevant mutations in melanoma and NSCLC [34].

Finally, the feasibility of genotyping pericardial effusion supernatant, when a drainage is necessary, has also been reported in 3 patients for the detection of *EGFR* driver [35] or T790M resistance [36] mutations.

## 5. Urine

Urine also contains free-floating tumor DNA and is particularly easy to collect repeatedly, giving it great potential for longitudinal follow up throughout treatment. Two fractions of urinary cfDNA have been described: high molecular weight nucleic acids originating from urinary tract and endothelial cells, and low molecular weight [50–250 base pairs] circulating DNA fragments excreted into urine following glomerular filtration by the kidneys. Urine cfDNA, derived from local tumor shed, is already an attractive tool to monitor the molecular profile of urological malignancies [37]. In other cancers, urine cfDNA, can serve as an alternative to plasma genotyping. In 63 patients with advanced *EGFR*-mutant NSCLC, sensitivities of tissue, plasma and urine were 73%, 82%, and 75%, respectively for T790M detection, these specimens being complementary [38]. In the same study, a significant decrease in T790M MAF in urine was observed in 9 patients treated with rociletinib, highlighting a potential for follow-up (cfDNA being extracted from the same volume). These findings were confirmed in another study investigating ctDNA kinetics in 8 patients treated with osimertinib, in whom the early kinetics of ctDNA shed into the urine correlated with tumor response [39]. Another prospective study in advanced NSCLC demonstrated a good correlation and complementarity between genomic profiles of cfDNA extracted from plasma, sputum, and urine compared to tissue [40]. In earlier stages, the analysis of DNA methylation at cancer-specific loci [41] in urine could help characterize nodules after screening via computed tomography (CT).

## 6. Saliva and Sputum

Like urine, saliva is another great example of an easy-to-access and potentially cost-effective alternative biospecimen. Data suggest it could be adequate for genotyping [42]. In advanced diseases, *EGFR* mutation detection is feasible in saliva [43], but with low sensitivity (46.2%) likely due to a low tumor content in tumors not proximal to airways [44].

A study combining five transcriptomic markers and a miRNA signature to discriminate cancer patients (*n* = 32) from controls (*n* = 64) reported a sensitivity of 94% but with a specificity of 83% [45]. This approach could be useful to help characterize nodules and guide potentially harmful biopsy procedures.

Sputum might be enriched with tumor-derived material compared to saliva and could help with lung cancer prediction in patients with a positive CT screen, using DNA hypermethylation of genes of interest [54]. The sensitivity and specificity of the combination of three genes (*TAC1*, *HOXA17*, and *SOX17*) in sputum was 93% and 89%, respectively, with a corresponding ROC AUC of 0.89 (95% CI: 0.80–0.98). In a validation cohort from the NELSON trial (subsequently diagnosed with cancer vs. control patients), this targeted methylation analysis approach remained specific (99.3%) but sensitivity was very low (17% for *RASSF1A*, 28% for the panel *RASSF1A*, *3OST2*, and *PRDM14*) [55]. Combining CT lung cancer screening and DNA methylation marker research on CT positive patients would relieve the problem of false positive scans and the unnecessary invasive procedures that result [46].

## 7. Cerebrospinal Fluid (CSF)

The cerebrospinal fluid (CSF) is another challenging cytology specimen. Exploiting CSF-derived cell-free DNA (CSF-cfDNA) is particularly appealing to avoid invasive surgical biopsies in primary brain tumors, but also in cases of brain metastases, because the number of tumor cells in CSF is usually low and DNA shed from central nervous system (CNS) tumors into the blood can be limited. Additionally, due to the blood brain barrier, the proportion of ctDNA is much higher in CSF than in blood, because normal DNA is sparsely released in CSF. The detection of CSF-cfDNA is feasible, more likely in cases of tumors adjacent to CSF reservoirs [47,48,49]. In NSCLC, *EGFR* mutation detection in CSF-cfDNA by ARMS-PCR is feasible (sensitivity 67%) but limited by a suboptimal concordance with tissue (specificity 82%) [50]. Through iterative hybrid-capture NGS of CSF-cfDNA, De Mattos-Aruda et al. demonstrated a good correlation between tumoral DNA load kinetics in CSF and response to local or systemic therapy in 6 patients, including 2 with NSCLC [48]. In contrast to the cytology study of CSF, the analysis of CSF-cfDNA could be very useful to identify molecular mechanisms of resistance to tyrosine kinase inhibitors (TKI) from a progression solely due to a poor central nervous system penetration of the drug. In patients with *ALK*-rearranged NSCLC with leptomeningeal carcinomatosis, liquid biopsy of CSF is more sensitive than plasma cfDNA to detect driver or resistance mutations and monitoring tumor response [51], even though repeating lumbar punctures seems invasive. CSF-cfDNA provided a comprehensive profiling of driver and resistance genes in 26 patients with leptomeningeal carcinomatosis. Driver genes were detected, by NGS, in 100%, 84.6%, and 73.1% of CSF-cfDNA, CSF precipitates, and plasma, respectively. Overall, 92.3% of patients had a much higher MAF in CSF-cfDNA compared to cells and plasma [52]. Another study of 26 NSCLC patients demonstrated a higher sensitivity of CSF-cfDNA compared to cytology (100% vs. 71%) for the diagnosis of leptomeningeal carcinomatosis [56].

## 8. Discussion, Perspectives, and Conclusions

The sensitivity of plasma genotyping can be limited, in particular when tumor burden is limited, and tissue biopsy in these patients is also challenging. Two approaches could address this issue: (i) considering other body fluids that contain tumor-derived free floating DNA at high concentrations and that outperform cytology for most of these pauci-cellular samples, with cfDNA being detected in cytologically negative samples [34]; (ii) improving methods to maximize the yield of biopsy procedures because invasive biopsy remains an integral part of any diagnostic strategy. The supernatant of cytology specimens is also a rich source of immediately available tumor DNA that appears to be highly complementary with tissue.

The interest of non-blood liquid biopsy is thus increasing, and multiple studies have demonstrated that cfDNA extracted from diverse body fluids can be a reliable source of genomic information, obtained consistently in a far less invasive manner than tissue biopsy.

Free-floating DNA in easy-to-access liquids, such as urine, sputum or bronchoscopy cytology samples’ supernatant could be very useful tools to discriminate malignant from benign nodules in the upcoming lung cancer screening era. CT scan is very sensitive but of poor specificity and, in addition, most blood-based screening tests have shown low sensitivity [6,10,57]. Exploiting liquids in direct contact to the tumor could represent a more reliable approach, even though most data regarding these biospecimens concern advanced stages.

In more advanced disease, body fluids collected near the tumor or metastases are more concentrated in cfDNA compared to blood, offering increased amounts of genetic material. A superiority of non-blood free DNA over cytology has not been clearly demonstrated but suggested by some reports, where ctDNA is detected in the supernatant of apparently negative cytology specimens (EBUS-TBNA [15] or pleural effusion [25]). Both are probably highly complementary, the limited cell block being preserved for diagnosis, and the immediately available supernatant for genomics. These high MAF may decrease the risk of false positive calls due to potential sequencing artefact of NGS. CfDNA may thus increase the overall yield of cytology specimens for tumor genotyping. Moreover, the turnaround time could be dramatically shortened, as free DNA is immediately available compared to the conventional approach where DNA can only be extracted after time-consuming diagnosis steps on FFPE blocks. This will be particularly interesting in the context of acquired resistance to targeted therapy where genomics is much more needed than histology. Cell -free DNA has also shown to be useful for prognostication, either by the analysis of tumor mutation burden [58] or by targeting alterations known to be associated with response or resistance to these agents [59] and to assess response to immune checkpoint inhibitors [59,60,61]. These potential applications could be extrapolated to the non-blood approaches described here.

In addition, these biospecimens could improve specificity by avoiding the detection of mutations linked to clonal hematopoiesis that can be detected in blood [62].

In conclusion, liquid biopsy should not be limited to blood. Tumor-derived materials are released in many other fluids in higher amounts when in direct contact with the tumor. Non-blood free-floating DNA could be of high value at all lung cancer stages, from screening and nodule characterization to genotyping patients with metastatic disease both at diagnosis and progression. Commercially available and clinically validated plasma genotyping platforms must be tested in the context of these additional samples before this approach can be translated into routine clinical use, as most of the studies reported here were performed with “in-house” sequencing platforms within academic research programs.

## Figures and Tables

**Table 1:** Diagnostic accuracy, advantages and potential future applications of free-floating DNA derived from body fluids.

Sample	State of the Art Advantages	Challenges Potential Future Applications
Plasma [5,6,7,8,9]	Non-invasive, rapid Sensitivity 70 to 80%, below 50% for stage I Early detection of acquired resistance (e.g., *EGFR* T790M) Addresses challenges of tumour heterogeneity	Integration of plasma NGS in routine clinical care for initial and resistance genotyping Minimal Residual Disease/Follow up Improve sensitivity in early diagnosis/Screening
FNA supernatant [12,13,14,15,16,17]	Rich and immediate source of tumor DNA Increases the yield of a low tumour content biospecimen for genomics and maybe diagnosis	Prospective validation of correlation with tissue, improvement in turnaround time and genomics feasibility Ultimately use supernatant for genomics and save cell block for pathology Improve the yield of bronchoscopy for nodule characterization
Brushing washing [18,19,20,21,22]	Increases the yield for cancer diagnosis Sensitivity of 88% for *EGFR* detection Prescence of lung cancer biomarkers	Fast discrimination between inflammatory pneumonitis (after radiation therapy, under immune therapy) and carcinomatous lymphangitis Lung cancer screening and nodule characterization (cfDNA, met-cfDNA, miRNAs)
Pleural and ascitic fluids [23,24,25,26,27,28,29,30,31,32,33,34]	Minimally invasive Good ratio tDNA/fDNA Reliable correlation with cell pellet Low turnaround time compared to tissue Increases the yield of low tumour content biospecimens	Integration of PE-ctDNA NGS in routine for initial and resistance genotyping Fast discrimination between benign and malignant PE (e.g., after surgery)
Urine [35,36,37,38,39,40]	Non-invasive, easy to collect Sensitivity around 70% Low turnaround time Can address tumour heterogeneity if trans-renal clearance of cfDNA (trDNA) Complimentary with plasma and tissue (T790M)	NGS: Wider range of genotype coverage Monitoring of response under targeted therapy Lung cancer screening and nodule characterization (cfDNA, met-cfDNA, miRNAs)
Saliva/sputum [41,42,43,44,45]	Non-invasive, easy to collect Potential for early lung cancer detection (miRNA, methylation) low sensitivity of *EGFR* mutation detection (42% for EGFR in sputum)	Lung cancer screening and nodule characterization (cfDNA, met-cfDNA, miRNAs)
Cerebrospinal fluid [46,47,48,49,50,51,52]	Minimally invasive High ctDNA MAF Increases the yield of a low tumour content biospecimen: diagnosis of leptomeningeal carcinomatosis (LC) in pauci or acellular samples	Discrimination between relapse and post-radiation necrosis after radiation therapy for brain metastasis Resistance analysis in CNS progression and distinction with poor drug penetration in CNS Monitoring of response for metastases and LC

BW: bronchial washing, PE: pleural effusion, CSF: cerebrospinal fluid, LC: leptomeningeal carcinomatosis, EBUS-TBNA: endobronchial ultrasonography transbronchial needle aspiration, NGS: next generation sequencing, cfDNA: cell free DNA, ctDNA: circulating tumor DNA, CNS: Central Nervous System, met-cfDNA: methylated cfDNA, trDNA: transrenal DNA.
